# Demographics of dogs, cats, and rabbits attending veterinary practices in Great Britain as recorded in their electronic health records

**DOI:** 10.1186/s12917-017-1138-9

**Published:** 2017-07-11

**Authors:** Fernando Sánchez-Vizcaíno, Peter-John M. Noble, Phil H. Jones, Tarek Menacere, Iain Buchan, Suzanna Reynolds, Susan Dawson, Rosalind M. Gaskell, Sally Everitt, Alan D. Radford

**Affiliations:** 1 Institute of Infection and Global Health, University of Liverpool, Waterhouse Building (2nd Floor, Block F), 1-5 Brownlow Street, Liverpool, L69 3GL UK; 2 Health Protection Research Unit in Emerging and Zoonotic Infections, National Institute for Health Research, University of Liverpool, Liverpool, UK; 3 Institute of Veterinary Science, Leahurst Campus, University of Liverpool, Chester High Road, Neston, CH64 7TE UK; 4 Institute of Infection and Global Health, Leahurst Campus, University of Liverpool, Chester High Road, Neston, CH64 7TE UK; 5 Health e-Research Centre (Farr@HeRC), Farr Institute, University of Manchester, Vaughan House, Portsmouth St, Manchester, M13 9GB UK; 6 British Small Animal Veterinary Association, Waterwells Business Park, Woodrow House, 1 Telford Way, Quedgeley, Gloucestershire, GL2 2AB UK

**Keywords:** Demographics, Companion animals, Electronic health records, Socioeconomic factors, SAVSNET

## Abstract

**Background:**

Understanding the distribution and determinants of disease in animal populations must be underpinned by knowledge of animal demographics. For companion animals, these data have been difficult to collect because of the distributed nature of the companion animal veterinary industry. Here we describe key demographic features of a large veterinary-visiting pet population in Great Britain as recorded in electronic health records, and explore the association between a range of animal’s characteristics and socioeconomic factors.

**Results:**

Electronic health records were captured by the Small Animal Veterinary Surveillance Network (SAVSNET), from 143 practices (329 sites) in Great Britain. Mixed logistic regression models were used to assess the association between socioeconomic factors and species and breed ownership, and preventative health care interventions. Dogs made up 64.8% of the veterinary-visiting population, with cats, rabbits and other species making up 30.3, 2.0 and 1.6% respectively. Compared to cats, dogs and rabbits were more likely to be purebred and younger. Neutering was more common in cats (77.0%) compared to dogs (57.1%) and rabbits (45.8%). The insurance and microchipping relative frequency was highest in dogs (27.9 and 53.1%, respectively). Dogs in the veterinary-visiting population belonging to owners living in least-deprived areas of Great Britain were more likely to be purebred, neutered, insured and microchipped. The same association was found for cats in England and for certain parameters in Wales and Scotland.

**Conclusions:**

The differences we observed within these populations are likely to impact on the clinical diseases observed within individual veterinary practices that care for them. Based on this descriptive study, there is an indication that the population structures of companion animals co-vary with human and environmental factors such as the predicted socioeconomic level linked to the owner’s address. This ‘co-demographic’ information suggests that further studies of the relationship between human demographics and pet ownership are warranted.

**Electronic supplementary material:**

The online version of this article (doi:10.1186/s12917-017-1138-9) contains supplementary material, which is available to authorized users.

## Background

Individuals within a pet population vary according to a wide range of characteristics including age, sex, species and breed. Since the species and breed of each individual animal are largely under the control of the owners, this variation is likely to be heavily impacted by human behaviour. Understanding demographic variation is critical to reducing disease risk and predicting the possible effects of interventions, and increasingly to the design of personalised health plans [1].

Demographic data may be available in some countries where it is required by regulators. However, in the absence of legislation, data are often lacking, and where present, driven by market forces. This is the case for companion animals in many countries, where there is no compulsory registration and little statutory disease notification. The companion animal sector is highly independent of government and whilst there is undoubtedly a wealth of demographic data generated, it is often fragmented in local databases and therefore not readily available for analysis [1]. Primary data collections can be made, but they are costly and time-consuming to establish and maintain.

Information on population demographics in the small animal sector has generally been obtained using cross-sectional surveys linked to specific studies [1–5]. Cohort studies could provide deeper epidemiological insights, as they often do in human health [6, 7]. However, data from companion animal cohorts are only now starting to become available [8, 9].

As a result, others have sought to harness existing databases such as pet health insurance data, microchipping, and pedigree registers which may be more accessible and cost effective, but as they only represent certain subpopulations they are prone to bias. Insurance databases can be useful for longitudinal studies [10, 11], but their data are generally only on diseases that result in claims [12]. Similarly, microchipping and pedigree registers do not represent the general population, although this situation is changing for dogs as microchipping has recently become compulsory in the UK [13].

Evidence suggests that in countries with developed pet industries, a high proportion of owned pet animals attend a veterinary surgeon [1, 14]. Asher et al. [1] estimated that 77% of the owned dogs in the UK were registered with veterinary practices and argued that surveys of veterinary practices could be useful in estimating the demographics of the owned dog population.

As health records become digitised they become more available for research [15]. In 1999, Lund et al. [14] used such records to explore population demographics in the USA. However, the records were manually supplemented with additional questionnaire data by practitioners and often data were available for only a small proportion of the sampled population. In England, O’Neill et al. [16] successfully collected electronic health records (EHRs) from a large population of animals; however, most of the practices were from only two regions restricting national generalisability.

SAVSNET, the Small Animal Veterinary Surveillance Network collects anonymised EHRs in real time from veterinary surgeons in practice and from commercial diagnostic laboratories throughout the UK, making them available for research [17, 18]. Data supply has been maintained by limiting the additional workload of participating practices and providing near-real-time benchmarking to data providers.

The objective of this study was to use EHRs collected over a full year by SAVSNET to describe the demographics of a diverse veterinary-visiting population of small companion animals across England, Scotland and Wales. In addition, we explored associations between a range of animal characteristics, including preventive health care interventions (such as neutering and insurance), and the socioeconomic status relative to the location of its owner. The methodology described means the results presented could be efficiently updated to monitor future trends over time.

## Methods

### Data collection

**Fig. 1:**
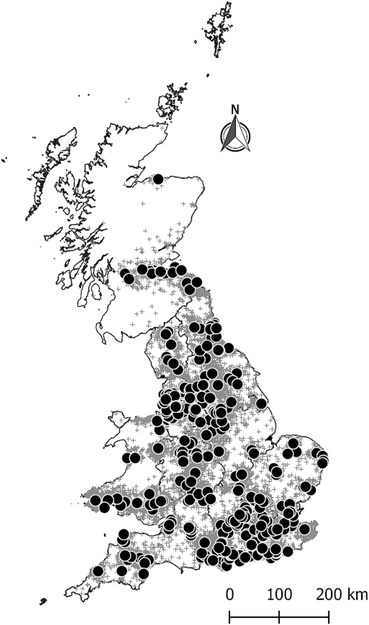
Geographical distribution of veterinary premises (*N* = 329; *black circles*) and animals (grey crosses) of the study. The boundaries for Great Britain depict the regions considered in the study (i.e. the countries of Scotland and Wales and the English regions of East Midlands, East of England, London, North East, North West, South East, South West, West Midlands, Yorkshire and The Humber). The reference map layers used contain: National Statistics data © Crown copyright and database right 2011 and 2012, NRS data © Crown copyright and database right 2011 and Ordnance Survey data © Crown copyright and database right 2011 and 2012

Data were collected electronically in near real-time from volunteer veterinary practices using a compatible version of practice management system (PMS) namely RoboVet (Vetsolutions, Edinburgh) and Teleos (Birmingham). Practices using these PMSs were approached and those expressing a willingness to participate in SAVSNET during a phone call were recruited. A ‘practice’ is defined as a single veterinary business, whereas ‘premise(s)’ includes all branches that make up a practice. This cross-sectional study uses a year of data from 143 of these practices (329 premises), chosen because they submitted uninterrupted data between 1st November 2014 and 31st October 2015, and represented 91.7% of total practices recruited by SAVSNET at the end of the study period and around 5.6% of UK veterinary practices (denominator from [1]). One hundred and twenty-four practices (295 premises) were recruited from England, eight practices (17 premises) from Scotland and 11 practices (17 premises) from Wales (Fig. 1). The EHRs were collected from consultations where a booked appointment was made to see a veterinary surgeon or nurse, and include the date the animal was seen, anonymous identifiers for each practice, premise and animal, the animal signalment (including species, breed, sex, neutering status, date of birth, date of neutering, insurance and microchipping status) and full owner’s postcode.

Owners attending practices participating in SAVSNET are informed about the project by a waiting room poster; those wishing to opt out are invited to tell their practitioner, who can then exclude all their data from the study. These opted out consultations are quantifiable for each practice, but no further data are captured by SAVSNET.

The collection and use of these data was approved by the University of Liverpool’s Research Ethics committee.

### Data management

The text-based data were cleaned for species and breed to deal with misspellings or the use of non-standard terms by mapping to standard terms. This was a two stage process of discovering the non-standard terms then developing/applying mapping rules. For example, to map the breed names (particularly dogs, cats and rabbits) a standard list of the most common breed names was taken from a reliable source (e.g. the UK Kennel Club for dog breeds). Each non-standard breed name in the clinical record was mapped to the standard name manually on its first occurrence. Further occurrences would then be matched automatically. Many breeds were present in the data set, some represented by only a few individuals, limiting further breed analysis. Thus, for the purposes of this study, only the animal’s breed, classified as purebred or crossbred, was further assessed.

Information from multiple visits for individual animals was included in the final analyses as follows. For animals attending veterinary practices on more than one occasion their age was calculated as the median age of all animal-age observations. These animals were considered to be neutered and/or insured and/or microchipped if these parameters were positively recorded on at least one consultation. Age at which an animal was neutered was calculated using the date of birth and the date of neutering when both parameters were captured. After examining and removing the outliers from the age profile of each species, the upper age limit for dogs, cats and rabbits was established as 24.5, 26 and 15.5 years old respectively.

Postcodes of owners were used to link each animal to the National Statistics Postcode Directory [19] and information concerning geographic location, i.e. country, region, Lower layer Super Output Area (for England and Wales) and datazone (for Scotland) classification. Regions in Great Britain were defined using level 1 of the Nomenclature of Units for Territorial Statistics (NUTS) which includes the countries of Scotland and Wales and the English regions of East Midlands, East of England, London, North East, North West, South East, South West, West Midlands, Yorkshire and The Humber (Fig. 1). The postcodes were also used to match each animal against databases containing Index of Multiple Deprivation (IMD) ranks for England 2010 [20], Scotland 2012 [21] and Wales 2011 [22]. A detailed description of how each government has developed their own measure of deprivation can be found elsewhere [23–25]. As a consequence IMD measures between these countries are not directly comparable. In England and Wales, the ranks of the Index are calculated for each Lower layer Super Output Area, whilst in Scotland these are calculated per each datazone. Ranks of the IMD for England, Wales and Scotland were independently categorised based on quintile cut-off scores with category 1 being least deprived and category 5, the most deprived.

### Statistical analysis

Descriptive statistics were used to characterise key demographic variables of this particular veterinary-visiting pet population and therefore statistical analyses were only required where specific associations between the exposure(s) and outcome(s) of interest were evaluated.

The asymptotic, linear-by-linear association test allows testing of the independence of two factors in case either both or one factor are ordered factors (i.e. ordinal variable) stratified by a third factor. A general description of this method is given by Agresti [26]. This method implemented in the R package ‘coin’ was performed to test whether there was a significant association between species (i.e. dogs and cats) and the age at which animals are presenting to SAVSNET veterinary practices. The continuous age variable was categorised as young (<1 year old), adult (1 to <8 years old) or aged (≥8 years old) as previously [17]. Age was considered in the test as an ordinal variable and the analysis was stratified by practice. The same analysis was conducted to assess whether there was an association between breed and age in dogs and cats. Statistical significance was defined as *P* < 0.05.

Mixed effects binary logistic regression models, incorporating veterinary practices as random effects, were used to assess the strength of association between the fixed effect IMD and several outcome variables such as dog ownership, cat ownership, breed ownership, two sex-neutering binary variables (with one being the neutering status in males and the other the neutering status in females), insurance status and microchipping status of dogs and cats. The association between IMD and each of these two sex-neutering binary variables was assessed using individual models. Separate models were undertaken for animals living in England, Scotland and Wales. Regression models were not conducted for rabbits because they are underrepresented in many veterinary practices as well as in categories of the explanatory variable and outcome variables, specifically in Wales and Scotland. The models were fitted using the Gauss-Hermite quadrature method with ten quadrature points per scalar integral implemented in the R package ‘lme4’. Statistical significance was defined as *P* < 0.05.

Statistical analyses were carried out using R language (version 3.0.1) [27].

## Results

### General demographic statistics

#### Number of animals and age profile of dogs, cats and rabbits

Data from 526,431 individual consultations were recorded, which represented 77.7% of total consultations including those where the client had opted out of study participation. When repeated consultations were removed, this included 186,044 unique dogs (64.8%), 86,995 cats (30.3%), 5626 rabbits (2.0%), 4684 other species (1.6%), and 3891 unmapped species (1.3%), the latter including 41% of animals where the species was originally unknown or not recorded. The geographical distribution of all animals included in the current study is presented in Fig. 1. The mean number of consultations per animal during the study period was 2.0, 1.6, 1.6 and 1.4 for dogs, cats, rabbits and other species respectively.

**Fig. 2:**
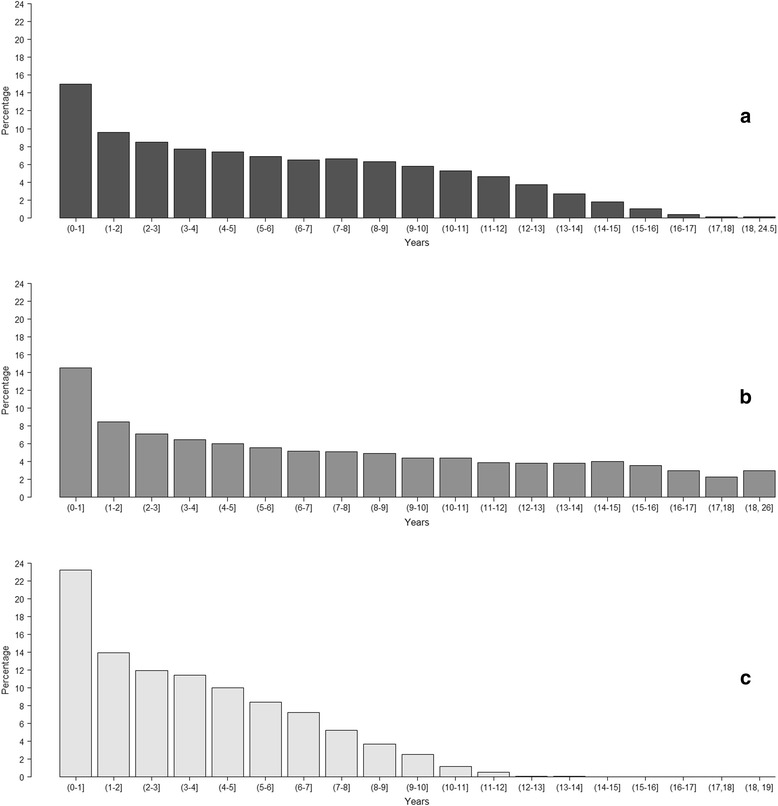
Age profile of dogs (**a**), cats (**b**) and rabbits (**c**) presenting to SAVSNET veterinary practices

The age profile of dogs, cats and rabbits presenting to SAVSNET veterinary practices is shown in Fig. 2. The percentage of dogs, cats and rabbits in which the date of birth was not recorded was 1.3% and the percentage in which it was considered not accurate was less than 0.01%. The median age, based on the median age of all an animal’s individual age observations, was 5.2 years in dogs (minimum value - maximum value: 0–24.5 years; interquartile range: 7.0 years), 6.2 in cats (0–26.0; 9.4) and 3.0 in rabbits (0–15.5; 4.4). The proportion of dogs and cats attending to SAVSNET veterinary practices during the study period was not the same for the three age categories (*χ* ^2^_df=1_ = 1237.7; *P* < 0.001), with a greater proportion of cats presenting when over 8 years of age than dogs (Table 1).

**Table 1:** Age profile of the veterinary-visiting population of dogs and cats in this study

Species	Breed	Number (percentage) of animals by age category		
		< 1 year	1 < 8 years	> = 8 years
Dog	Total	27,828 (15.1)	97,929 (53.1)	58,758 (31.8)
Dog	Purebred	19,522 (14.4)	72,146 (53.2)	43,922 (32.4)
Dog	Crossbred	4067 (15.9)	13,033 (50.9)	8510 (33.2)
Cat	Total	12,567 (14.8)	37,374 (43.8)	35,324 (41.4)
Cat	Purebred	1260 (15.5)	3794 (46.5)	3095 (38.0)
Cat	Crossbred	9648 (13.9)	30,216 (43.6)	29,413 (42.5)

The Table shows the number and percentage of total number of animals by species (i.e. dogs and cats), breed and by age category. For both species, the number of purebred and crossbred animals does not sum up to the total number of animals because a mapped breed was not available for all individuals.

#### Number of animals by breed in dogs, cats and rabbits, and age profile by breed in dogs and cats

**Fig. 3:**
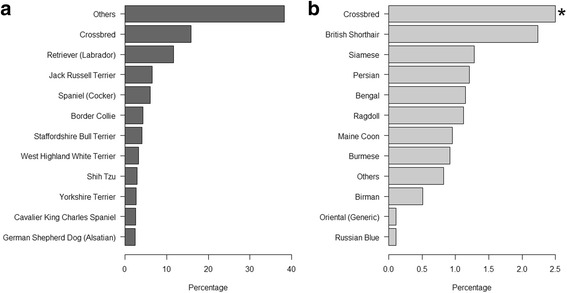
Percentage of total dogs by dog breed (**a**) and total cats by cat breed (**b**). The asterisk (*) indicates that the percentage of total cats represented by crossbred cats was limited for presentation purposes because they represented such a large proportion of the population; crossbred cats accounted for 89.6% of total cats in this population

A mapped breed was available for 87.7% of all dogs, cats and rabbits. The remainder included animals where the breed recorded comprised a large number of rare misspellings as well as animals where the breed was either unrecorded or not recognised by the practitioner. Where a mapped breed was available, 84.1% of dogs and 98.2% of rabbits were recorded as purebred. This was in stark contrast to cats where the figure was much lower (10.4%). The 10 most popular purebreds accounted for 74,648 dogs (45.9%) and 7634 cats (9.6%) (Fig. 3). Labrador Retriever (11.6%) and British Shorthair (2.2%) were the most popular breeds of dog and cat, respectively.

The proportion of crossbred cats and purebred cats attending to SAVSNET during the study period was not the same for the three age categories (*χ* ^2^_df=1_ = 61.6; *P* < 0.001), with a greater proportion of crossbred animals presenting over 8 years of age (Table 1). This relationship between purebred status and age was not significant in dogs (*χ* ^2^_df=1_ = 0.5; *P =* 0.5).

#### Number of animals by sex in dogs, cats and rabbits

In the veterinary-visiting population assessed, there were approximately equal numbers of female and male dogs and cats with females making up 49.3% of dogs and 51.9% of cats. The same was true in each species at breed level, with females making up 49.1% of purebred dogs, 50.1% of crossbred dogs, 48.2% of purebred cats and 52.1% of crossbred cats. In rabbits, there was some deviation from this with females making up 43.7% of all rabbits, 41.8% of recorded purebred rabbits and 30.0% of recorded crossbred rabbits.

### Key performance indicators (KPIs) statistics

#### Neutering status

Over half of dogs were neutered (57.1%), including 55.0% of males and 59.2% of females. In this veterinary-visiting population neutering was more common in cats (77.0%), including 78.4% of males and 75.8% of females. Less than half of the rabbits were neutered (45.8%), including 50.0% of males and 40.3% of females.

In this SAVSNET study population the neutering relative frequency was higher in male crossbred dogs (62.5%) than in male purebred dogs (53.43%) and in female crossbred dogs (65.1%) than in female purebred dogs (58.6%). In cats, the percentage of neutered animals was slightly higher in male purebreds (80.1%) than in male crossbreds (79.1%) and in female crossbreds (77.4%) than in female purebreds (75.3%). In rabbits, the neutering relative frequency was higher in male purebreds (52.7%) than in male crossbreds (28.6%) and higher in female crossbreds (50.0%) than in female purebreds (41.3%).

**Fig. 4:**
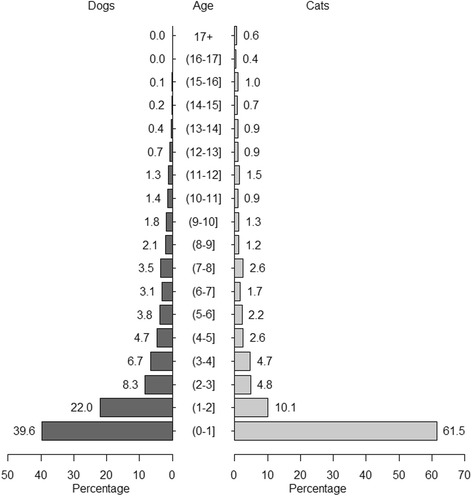
Age (in years) at time of neutering in dogs and cats. The percentage of dogs and cats neutered at a given age, for the 51.2% of neutered dogs and 42.3% of neutered cats where this age was known

**Fig. 5:**
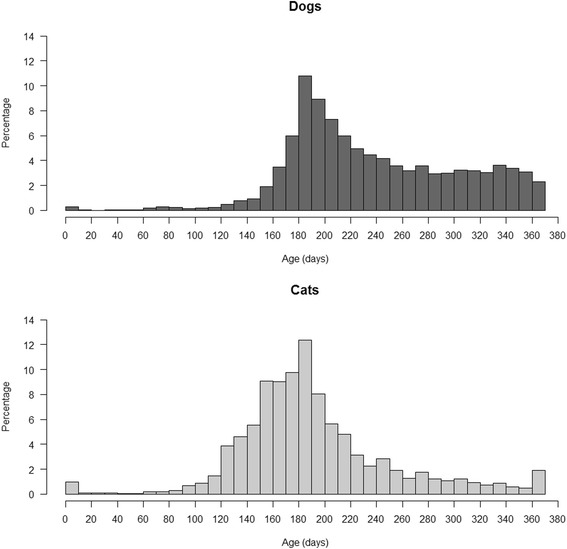
Age at time of neutering for dogs and cats neutered in their first year of life. The percentage of dogs and cats neutered is shown for 10 day intervals

The age of neutering was recorded in 51.2% of neutered dogs and 42.3% of neutered cats. For these animals, the recorded age at neutering is shown in Fig. 4, with 39.6% of neutered dogs and 61.5% of neutered cats recorded as being neutered within their first year of life. Fig. 5 shows a higher age resolution of the percentage of neutered dogs and cats in their first year of life, suggesting that in both species, neutering peaks at around 180 days of age. This equates to only 0.7 and 6.9% of all neutered dogs being neutered within the first four and 6 months of life respectively. In cats, these percentages were higher, with 3.3 and 30.5% of all neutered cats being neutered within the first four and 6 months of life respectively.

#### Insurance and microchipping status

The recorded relative frequency of insurance for dogs, cats and rabbits was 27.9, 18.5 and 9.1%, respectively. The recorded percentage of insured animals was slightly higher in purebred dogs (28.4%) than in crossbred dogs (26.8%) and higher in purebred cats (24.7%) than in crossbred cats (18.2%).

More than half of dogs (53.1%) were recorded as being microchipped, whilst only 39.9% of cats and 4.4% of rabbits were microchipped. Like insurance, in this SAVSNET study population the microchipping relative frequency was higher in purebred dogs (53.8%) than in crossbred dogs (50.7%) and higher in purebred cats (44.8%) than in crossbred cats (40.0%).

### Geographical area and practice

The main demographic outcomes obtained for this SAVSNET veterinary-visiting population are summarised at geographical and practice level in two additional files [see Additional files 1 and 2, respectively]. Rabbits were excluded from the results at practice level because they were underrepresented in a large number of practices.

### Socioeconomic status

In 94.6% of all animals where the owner had not opted out of study participation, a valid owners’ full postcode was recorded, which allowed them to be matched against national databases linking geographic location with IMD ranks.

#### Species and breed ownership

The distribution of animals by species, British country and IMD category is shown in an additional file [see Additional file 3]. Of the animals presented to these SAVSNET practices, the odds of the animal being a dog (compared to non-dogs) were significantly lower if its owner was living in lesser deprived areas of England, Wales and Scotland than the most deprived areas of these countries (England: *P* < 0.001 for IMD categories 4 and 1, *P* < 0.01 for IMD 3, and *P* < 0.05 for IMD 2; Wales: *P* < 0.001 for IMD categories 3 and 2, and *P* < 0.05 for IMD 4; Scotland: *P* < 0.01 for IMD 1, and *P* < 0.05 for IMD 2) [see Additional file 4 for detailed statistical output]. The reverse association was true in cats [Additional file 4].

Of the dogs attending SAVSNET practices, the odds of the animals being purebred were significantly higher if their owners were living in lesser deprived areas of England rather than the most deprived areas of the country (*P* < 0.001 for IMD categories 1–3) [Additional file 4]. In Wales and Scotland, this association was only significant in IMD category 2 (*P* < 0.01) and IMD category 1 (*P* < 0.05) [Additional file 4], respectively. For cats, the same association was significant in England for IMD categories 1–3 (*P* < 0.001) and it was not significant in Wales and Scotland [Additional file 4].

#### Neutering, insuring and microchipping status

A significant relationship was found between being neutered, insured or microchipped and the predicted IMD based on the location of the pet owner [see Additional file 5 for detailed statistical output]. Of the male and female dogs attending SAVSNET practices, the odds of being neutered were significantly higher if their owners were living in lesser deprived areas of England, Scotland and Wales rather than the most deprived areas (male: *P* < 0.05 for IMD categories 1–4 in England and Wales, and IMD 1–3 in Scotland; female: *P* < 0.05 for IMD 1–4 in England, Wales and Scotland). The same association was found for male and female cats in England (*P* < 0.05) and male cats in Wales in IMD categories 1–3 (*P* < 0.05) and for male and female cats in IMD category 1 in Scotland (*P* < 0.05) [Additional file 5].

For dogs, the odds of being insured and microchipped were significantly higher if their owners were living in lesser deprived areas of Great Britain rather than the most deprived areas (insurance: *P* < 0.05 for IMD categories 1–4 in England and for IMD 1–3 in Wales and Scotland; microchipping: *P* < 0.05 for IMD categories 1–4 in England and Scotland, and IMD 1–3 in Wales) [Additional file 5]. The same association was found for cats in England and Wales (*P* < 0.05) [Additional file 5], except for cats being microchipped in the IMD category 2 in Wales. In Scotland, this association was only significant for cats being microchipped in IMD category 1 (*P* < 0.05) [Additional file 5].

## Discussion

Demographic variables influence health and welfare in humans and animals through at least two interrelated phenomena, namely the population’s characteristics (its size and its composition by age, sex, species, breed, etc.) [28] and the characteristics of the environment in which a given population live (e.g. geographical distribution, socioeconomic factors). Application of disease control measures and possible interventions require an understanding of the demographic context and how it is changing over time. This study has used routinely collected EHRs from volunteer veterinary practices to describe key demographic variables of a large population of veterinary-visiting companion animals across Great Britain.

### Species presenting to practice

The proportion that dogs, cats, rabbits and other species represented in our population was consistent with comparable studies [18, 29]. There is now a confluence of data from disparate sources to suggest the numbers of owned cats and dogs are broadly similar in the UK. Based on a telephone survey in 2011, there were an estimated 11,599,824 dogs (95% CI: 10,708,070–12,491,578) and 10,114,764 cats (9,138,603–11,090,924) in the UK [30]. Although not peer reviewed, the Pet Food Manufacturers’ Association publishes generally accepted estimates of pet ownership with most recent figures for 2015 estimating 8.5 million dogs, 7.4 million cats and 1.0 million rabbits in 24.0, 17.0 and 2.0% UK households, respectively. It is therefore interesting that individual dogs both made up a 2.1-fold greater proportion of the veterinary-visiting population of this study than cats, and also that, on average, each individual dog attended a SAVSNET veterinary surgery 1.2 times more often than cats. This raises important clinical and social questions on how often individual cats and dogs get ill, how often they are recognised as being ill by their owners, and how motivated their owners are to seek veterinary care either when ill, or for other preventive health care when well.

### Association between postcode predictors of deprivation and species ownership

Because SAVSNET collects full owners’ postcodes, each EHR can be matched against published predictors of human socioeconomic deprivation. Here we show that regardless of predicted deprivation, dogs always made up the largest proportion of species presented to their veterinarian. However, the odds of the animal being a dog (compared to non-dogs) were significantly lower if its owner was living in lesser deprived areas of Great Britain than the most deprived areas of the country. The reasons for this are not clear, but it may be that owners in the most deprived areas take their dog to the veterinarian more often than they do in lesser deprived areas. Alternatively, dogs may comprise a larger proportion of species living in the most deprived areas than in lesser deprived, or owners may take other species to the veterinarian less often than they do in lesser deprived areas, or there may be a combination of such factors. Since most of non-dogs were cats, not surprisingly the reverse association was true in cats. Other studies have shown a similar association of socioeconomic factors and cat ownership in the UK and elsewhere. In a telephone survey, although household income itself was not significant, households containing one or more people with a university degree were 1.36 times more likely to own a cat than other households [3]. In the USA, cat ownership, as measured by the presence of cat allergens, was more common in households where the mother had a higher level of school education [31] and in areas with low levels of poverty [32]. The reasons for this correlation between socioeconomics and pet preference are likely to be complex. However, since they will impact on animal welfare and human health, they warrant further research.

### Breed status

Whilst the vast majority of dogs (84.1%) and rabbits (98.2%) in the veterinary-visiting population of this study were considered purebred, the opposite was the case for cats (10.4%). This is broadly consistent with previous studies in dogs in the UK [16, 29], and elsewhere [11, 14], and cats [14, 29, 33]. These findings reaffirm the desire of the public in Great Britain to own dogs of a recognisable breed, and may explain in part the highly developed and diverse dog breeding industry in the UK and elsewhere. Consistent with previous studies, the Labrador Retriever was the most common breed in this population [1, 16, 34]. Indeed the top six breeds reported here were the same as those described based on an entirely different practice data set (Sánchez-Vizcaíno F: Population demographics, unpublished).

### Age profile of species and breeds

Cats’ median age (6.2 years) was higher than dogs (5.2) and rabbits (3.0), demonstrated by a greater proportion of cats over the age of eight in the studied population. This was consistent with previous figures based on a smaller population of observed consultations [4]. Interestingly, a significant association was also found between breed and the age category at which cats attended SAVSNET veterinary practices, with a greater proportion of cats over the age of 8 years presenting in the crossbred group. The ages used are those at presentation to a veterinary surgeon or nurse and may be affected by many factors including an individual animal’s underlying susceptibility to disease, and socioeconomic factors of their owners. Therefore, further studies aimed to understand the patterns of morbidity with age as well as life expectancies in breeds of dogs and cats are required.

The dogs and cats in our study were somewhat older than those in previous similar studies based on EHRs by O’Neill et al. [16] (dogs 4.5 years) and Lund et al. [14] (dogs 4.8 years and cats 4.3 years). Whilst the reasons for this are unknown they may relate to differences between the sampled populations and how individual EHRs are collected; age in the current study was based on booked consultations whereas other studies may only require that animals are under veterinary care.

### Pet neutering status and age of neutering for each species

That neutering is an effective intervention to prevent unwanted pregnancy in companion animal species is without doubt. However, neutering in companion animals is used for many other reasons such as disease prevention and behaviour modification [35]. In our population, neutering was more common in cats (77.0%), than in dogs (57.1%) and rabbits (45.8%). These values for dogs and cats are broadly similar to those in other studies in the UK including cats ([9] – 73.5%) and dogs ([36] – 49.8%, [16] – 41.1%, [37] – 54.0%). Where both cats and dogs have been included in the same study the trend to neuter cats more than dogs is also conserved: in the UK [38], in Ireland [2], and in the USA [14]. Within species, there were also interesting differences in our population between the neutering of the sexes. For cats, males were more frequently neutered than females, possibly reflecting owner concerns around the behaviour of entire tomcats, and also the relative ease and lower cost of neutering in males. This trend was reversed in dogs, consistent with a survey showing veterinary surgeons were more likely to recommend neutering of female dogs than male dogs [37]. This pattern has also been observed in other studies [2, 4, 14], suggesting that fairly consistent underlying pressures are driving the neutering of pet animals in disparate populations (UK, Ireland, USA). Differences observed between the neutering frequencies of purebred and crossbred animals in all species are likely to result from complex interactions between owner demographics and intentions to breed.

Guidelines encourage neutering of cats soon after the first vaccinations are complete and at around 4 months-of-age [39, 40]. Neutering cats prior to sexual maturity is strongly recommended to prevent unintended litters, and to avoid neutering of female cats while they are pregnant [41]. Furthermore, cats neutered by 4 months of age were shown to have significantly lower complication rates [42] with shorter surgery duration, lower surgical morbidity rates and quicker recovery from anaesthesia compared with cats neutered at 6 months of age or older [43–45]. In our study population, based on the recorded neutering date and age, only approximately one third of neutered cats were recorded as being neutered within their first 6 months of life. This points to a significant proportion of cats where current guidelines may not be being followed, with potential impact on animal welfare, both directly, and through an increased risk of unwanted pregnancies.

Guidelines recommending the age of neutering are less definitive for dogs; according to the British Veterinary Association [35], there is insufficient data to form a position on the early neutering of dogs. Our study, showed that only 6.9% of neutered dogs were recorded as being neutered within their first 6 months of life.

Owner predicted deprivation was also associated with the neutering frequency. Both for male and female dogs attending SAVSNET practices, the odds of being neutered were significantly higher if their owners were living in lesser deprived areas of Great Britain than the most deprived areas of the country. The same association was found for male and female cats in England and male cats in Wales and a similar although less clear trend was seen for male and female cats in Scotland. Our previous pilot study based on data collected in a similar way but from a different PMS and different smaller populations distributed through England and Wales found similar results [46]. Within cats, factors including increased household income and obtaining their cat from a rescue organisation were positively associated with increased neutering by 6 months-of-age [9]. In future studies it will be critical to consider the health psychology underlying owner choices to neuter their pets.

### Pet insurance status for each species

The relative frequency of insurance in pets is generally considered to be relatively high in the UK compared to some other developed countries such as the USA and Canada where the estimates suggest that just 0.3–3.0 and 4.0% of dogs are insured, respectively (reviewed by O’Neill et al. [15]). In this study, the recorded percentage of insured animals was highest in dogs, 1.5 times greater than that of cats, and lowest in rabbits. These findings are similar to those based on a second different UK population in veterinary practices using a different PMS (Sánchez-Vizcaíno F: Population demographics, unpublished). Other studies however have shown insurance to be quite variable in dogs (19.0–40.3%) [1, 15, 36, 38]. This variation may be driven by differences in study population, methodology and timing. Of the dogs and cats presented to SAVSNET practices, with the exception of cats from Scotland, the odds of being insured were significantly higher if their owners were living in the least deprived areas than the most deprived areas, and consistent with our previous pilot study for England and Wales [46]. It seems probable that the insured population of animals is therefore quite different from the uninsured population, with likely impacts on the health of individual animals, as well as the veterinary health seeking behaviour and preventive health care taken by the owner. Studies of health burden based on insured animals may therefore not be generalisable to uninsured animals [12].

### Pet microchipping status for each species

Microchipping is one of the best ways to reunite lost or stolen pets with their owners and reduce the number of pets in shelters. In our population, we found that the recorded relative frequency of microchipping was higher in dogs than in cats and rabbits, and higher than that reported for dogs in an England-based study by O’Neill et al. [16]. This variation could be driven by differences in the sampled population. As shown by our previous pilot study for England and Wales [46], we confirm here that socioeconomic factors seem to be associated with this intervention. For the dogs and cats attending SAVSNET practices, the odds of being microchipped were significantly higher if their owners were living in the least deprived areas of Great Britain rather than the most deprived areas. Clearly the recent introduction of compulsory microchipping of dogs across the UK will radically change these proportions in the coming months and years. However, this legislation only covers the dog; it will be interesting to monitor the impact on other species as more dogs become microchipped. Compulsory microchipping also provides new resources to explore population demographics, where these can ethically be made available for research [1].

### Data limitations

All results are necessarily based on data as recorded in individual EHRs such that our observations may be impacted by the quality of data recording in individual animals and practices. EHRs are only available from those animals whose owners did not exclude their data by opting out. This study is cross-sectional in nature so the status of time variable exposures in the study population such as the veterinary practice the animal attends, the region the owner lives or the IMD relative to the location of the pet owner are ascertained only for the time in which the study is conducted. In these instances, the investigator cannot be certain that the exposure preceded the outcome (one of the fundamental criteria for establishing causation). Therefore, this kind of study can produce measures of association but cannot ‘prove’ causation.

Veterinary practices contributing data to this study were selected by convenience based on their use of a compatible version of PMS and recruited based on the willingness to take part in SAVSNET. Hence, prevalence of demographic parameters may be very different in this study population compared to those in the overall veterinary-visiting population of small animals across Great Britain (target population). However, any observed association between the exposure(s) and outcome(s) of interest is more likely to be generalisable, especially, to the source population [47] (i.e. the overall veterinary-visiting population of small animals attending veterinary practices using a SAVSNET compatible version of PMS across Great Britain). This is reinforced by the fact that the practices included in the current study were widely distributed around England, Wales and Scotland and represented 24.5% of those practices that contained the source population and approximately 5.6% of those practices that contained the target population in 2009 [1]. It is also of note that the dog population of this study represented an estimated 2.1% of the UK dog veterinary-visiting population (it would be higher for Great Britain) given the assumptions that the dog ownership was 11,599,824 [30] and that 77% of the owned dogs in the country were registered with veterinary surgeons [1]. Thus, despite the selection bias, the authors have identified and measured the strength of potential associations, highlighting areas of interest for future research.

It is also of note that the anonymous nature of both individual animal identification and individual owner identification means it is not possible for us to tell if the same animal is seen in different practices, nor whether more than one animal is owned by the same owner. So the potential effect of “owner” in an outcome of interest could not be assessed in our models. One limitation of using IMD is that it reflects the socioeconomic status relative to the pet owners’ location and not necessarily the current socioeconomic status of individual owners. IMD is a wider concept than poverty, and is calculated weighting different types of deprivations, or domains that might occur in each Lower layer Super Output Area of England and Wales and each datazone of Scotland. The ranks of some of the domains used in these three countries such as protection from crime, access to services and living environment are expected to be mostly given by the characteristics of the areas for which they are calculated regardless of the wealth of the individuals living in those areas. Thus, the authors believe that IMD could still be a valuable proxy for a general socioeconomic status of individual pet owners as people living in the same area necessarily share several types of deprivation.

The date of birth was not captured in 1.3% of dogs, cats and rabbits, and 5.4% of owners’ postcode, 1.3% of animal species and the breeds of 12.3% of animals were not mapped. This lack of information was considered small when compared with the study population and therefore one would expect that if it were recorded would not modify the overall conclusions obtained from this study. Conclusions from the age profile at time of neutering should be interpreted with caution because in almost half of neutered dogs and 57.7% of neutered cats this information was not recorded in the clinical record. It is also likely that the age of neutering was not always accurate as a small number of animals were recorded to be neutered just after they were born or at a very early age. However, these errors were considered negligible when they are seen in the context of the total study population (Fig. 5). It is also of note that species and breed classification of animals were as accurate as the practitioner’s criterion for its classification was. Univariable mixed effects logistic regression models were used to model the relationships between socioeconomic status and various KPIs such as neutering, insurance and microchipping. These associations were only assessed in the context of the veterinary-visiting population of small companion animals. Future analyses, including more explanatory variables like breed, age and sex would augment the current results, providing further understanding into owner and veterinary surgeon behaviour.

## Conclusions

Up-to-date demographic data are essential for understanding populations at risk, and for exploring the variations within populations and how these fundamental patterns relate to health. This study could only have been accomplished through the seamless collection and use of EHRs at scale from private veterinary practices. To the best of the authors’ knowledge, this is the first time that, by linking individual animals through postcodes to area-based estimates of material deprivation, socioeconomic factors have been investigated with regard to species ownership, breed ownership, microchipping status, and preventive health care interventions such as neutering and insurance in both dogs and cats throughout Great Britain. In the future, through ongoing collection and longitudinal analysis of these kinds of data, practitioners will be able to monitor and adapt local policies to their prevailing demographics.

## Additional files

Additional file 1: Demographics of the SAVSNET veterinary-visiting population of dogs, cats and rabbits by each region considered in the study. (DOCX 18 kb)

Additional file 2: Demographics of the SAVSNET veterinary-visiting population of dogs and cats summarised at practice level. (DOCX 13 kb)

Additional file 3: Number of animals stratified by species, British country and Index of Multiple Deprivation (IMD). Species assessed include dogs, cats and other species. IMD category 1 indicates the least deprived areas and category 5 the most deprived. The percentage that each species made up within each IMD category in each country is shown in brackets. (DOCX 13 kb)

Additional file 4: Results of the mixed effects logistic regression models, assessing the association between a range of an animal’s characteristics and the Index of Multiple Deprivation (IMD). Shown are odds ratios of fixed effects IMD in England, Wales and Scotland from the final mixed effects logistic regression models of; the probability of animals being a dog in the veterinary-visiting population; the probability of animals being a cat in the veterinary-visiting population; and of the probability of dogs and cats being purebred in the veterinary-visiting dog and cat population, respectively. Three asterisks (***), two asterisks (**) and one asterisk (*) indicate *p* < 0.001, *p* < 0.01 and *p* < 0.05, respectively. CI = confidence interval. (DOCX 15 kb)

Additional file 5: Results of the mixed effects logistic regression models, assessing the association between a range of an animal’s key performance indicators and the Index of Multiple Deprivation (IMD). Shown are odds ratios of fixed effects IMD in England, Wales and Scotland from the final mixed effects logistic regression models of; the probability of dogs and cats being neutered by sex; the probability of dogs and cats being insured; and of the probability of dogs and cats being microchipped. Asterisk (*) indicates *p* < 0.05. CI = confidence interval. (DOCX 19 kb)

## Abbreviations

CI: Confidence interval
EHRs: Electronic health records
IMD: Index of multiple deprivation
NUTS: Nomenclature of units for territorial statistics
PMS: Practice management system
SAVSNET: The small animal veterinary surveillance network

## References

[1] Asher L, Buckland EL, Phylactopoulos CI, Whiting MC, Abeyesinghe SM, Wathes CM (2011). Estimation of the number and demographics of companion dogs in the UK. BMC Vet Res.

[2] Downes M, Canty MJ, More SJ (2009). Demography of the pet dog and cat population on the island of Ireland and human factors influencing pet ownership. Prev Vet Med.

[3] Murray JK, Browne WJ, Roberts MA, Whitmarsh A, Gruffydd-Jones TJ (2010). Number and ownership profiles of cats and dogs in the UK. Vet Rec.

[4] Robinson NJ, Brennan ML, Cobb M, Dean RS (2015). Capturing the complexity of first opinion small animal consultations using direct observation. Vet Rec.

[5] Stavisky J, Brennan ML, Downes M, Dean R (2012). Demographics and economic burden of un-owned cats and dogs in the UK: results of a 2010 census. BMC Vet Res.

[6] Pearson H (2012). Children of the 90s: coming of age. Nature.

[7] Wright J, Small N, Raynor P, Tuffnell D, Bhopal R, Cameron N (2013). Cohort profile: the born in Bradford multi-ethnic family cohort study. Int J Epidemiol.

[8] Pugh CA, BMd B, Handel IG, Summers KM, Clements DN (2014). What can cohort studies in the dog tell us?. Canine Genet Epidemiol.

[9] Welsh CP, Gruffydd-Jones TJ, Murray JK (2013). The neuter status of cats at four and six months of age is strongly associated with the owners’ intended age of neutering. Vet Rec.

[10] Egenvall A, Bonnett BN, Olson P, Hedhammar A (2000). Gender, age and breed pattern of diagnoses for veterinary care in insured dogs in Sweden during 1996. Vet Rec.

[11] Egenvall A, Bonnett BN, Olson P, Hedhammar A (2000). Gender, age, breed and distribution of morbidity and mortality in insured dogs in Sweden during 1995 and 1996. Vet Rec.

[12] Egenvall A, Nodtvedt A, Penell J, Gunnarsson L, Bonnett BN (2009). Insurance data for research in companion animals: benefits and limitations. Acta Vet Scand.

[13] Anon. The Microchipping of Dogs (England) Regulations 2015. In: Parliament, H. o., (ed.). 2015.

[14] Lund EM, Armstrong PJ, Kirk CA, Kolar LM, Klausner JS (1999). Health status and population characteristics of dogs and cats examined at private veterinary practices in the United States. J Am Vet Med Assoc.

[15] O'Neill DG, Church DB, McGreevy PD, Thomson PC, Brodbelt DC (2014). Approaches to canine health surveillance. Canine Genet Epidemiol.

[16] O'Neill DG, Church DB, McGreevy PD, Thomson PC, Brodbelt DC (2014). Prevalence of disorders recorded in dogs attending primary-care veterinary practices in England. PLoS One.

[17] Jones PH, Dawson S, Gaskell RM, Coyne KP, Tierney A, Setzkorn C (2014). Surveillance of diarrhoea in small animal practice through the small animal veterinary surveillance network (SAVSNET). Vet J.

[18] Radford AD, Noble PJ, Coyne KP, Gaskell RM, Jones PH, Bryan JG (2011). Antibacterial prescribing patterns in small animal veterinary practice identified via SAVSNET: the small animal veterinary surveillance network. Vet Rec.

[19] Office for National Statistics. Postcode Products. In: Open Geography, Download products. 2014. http://geoportal.statistics.gov.uk/datasets?q=ONS%20Postcode%20Directory%20(ONSPD)&sort=name. Accessed 28 Oct 2014.

[20] Department for Communities and Local Government. English indices of deprivation 2010: indices and domains. In: Statistics, English indices of deprivation 2010. 2011. https://www.gov.uk/government/statistics/english-indices-of-deprivation-2010. Accessed 17 Oct 2014.

[21] The Scottish Government. Part 2 – SIMD 2012 Data – Overall ranks and domain ranks. In: Statistics, Scottish Index of Multiple Deprivation, SIMD 2012 Publication Web Portal, Download SIMD 2012 Data. 2012. http://simd.scotland.gov.uk/publication-2012/download-simd-2012-data/. Accessed 17 Oct 2014.

[22] Welsh Government. WIMD 2011 individual domain scores and overall index scores for each Lower Layer Super Output Area (LSOA). In: Statistics & Research, Welsh Index of Multiple Deprivation (WIMD), Past releases, 2011. 2011. http://gov.wales/statistics-and-research/welsh-index-multiple-deprivation/?lang=en#?tab=previous&lang=en&_suid=1434998682468013747584768796872. Accessed 17 Oct 2014.

[23] Department for Communities and Local Government. English indices of deprivation 2010. In: Statistics, English indices of deprivation 2010. 2011. https://www.gov.uk/government/statistics/english-indices-of-deprivation-2010. Accessed 17 Oct 2014.

[24] The Scottish Government. Overview of the SIMD. In: Statistics, Scottish Index of Multiple Deprivation, SIMD 2012 Publication Web Portal, Introduction to SIMD 2012. 2012. http://simd.scotland.gov.uk/publication-2012/introduction-to-simd-2012/overview-of-the-simd/what-is-the-simd/. Accessed 17 Oct 2014.

[25] Welsh Government. Welsh Index of Multiple Deprivation, 2011: Summary report. In: Statistics & Research, Welsh Index of Multiple Deprivation (WIMD), Past releases, 2011. 2011. http://gov.wales/statistics-and-research/welsh-index-multiple-deprivation/?lang=en#?tab=previous&lang=en&_suid=1434998682468013747584768796872. Accessed 17 Oct 2014.

[26] Agresti A (2002). Categorical data analysis.

[27] R Core Team. R: language and environment for statistical computing. In: R Foundation for Statistical Computing, Vienna, Austria. 2015. http://www.R-project.org/. Accessed 22 June 2015.

[28] Lopez AD, Begg S, Bos E, Lopez AD, Mathers CD, Ezzati M, Jamison DT, Murray CJL (2006). Demographic and epidemiological characteristics of major regions, 1990–2001. Global burden of disease and risk factors.

[29] Robinson NJ, Dean RS, Cobb M, Brennan ML (2015). Investigating common clinical presentations in first opinion small animal consultations using direct observation. Vet Rec.

[30] Murray JK, Gruffydd-Jones TJ, Roberts MA, Browne WJ (2015). Assessing changes in the UK pet cat and dog populations: numbers and household ownership. Vet Rec.

[31] Leaderer BP, Belanger K, Triche E, Holford T, Gold DR, Kim Y (2002). Dust mite, cockroach, cat, and dog allergen concentrations in homes of asthmatic children in the northeastern United States: impact of socioeconomic factors and population density. Environ Health Perspect.

[32] Kitch BT, Chew G, Burge HA, Muilenberg ML, Weiss ST, Platts-Mills TA (2000). Socioeconomic predictors of high allergen levels in homes in the greater Boston area. Environ Health Perspect.

[33] Murray JK, Gruffydd-Jones TJ (2012). Proportion of pet cats registered with a veterinary practice and factors influencing registration in the UK. Vet J.

[34] Kennel Club. Top 20 Breeds 2013–2014. In: Breed registration statistics. 2015. http://www.thekennelclub.org.uk/media/350279/2013_-2014_top_20.pdf. Accessed 4 June 2015.

[35] British Veterinary Association. Neutering of cats and dogs. In: News, campaigns and policy. Policy. Companion animals. 2015. http://www.bva.co.uk/News-campaigns-and-policy/Policy/Companion-animals/Neutering/. Accessed 5 June 2015.

[36] O'Neill DG, Church DB, McGreevy PD, Thomson PC, Brodbelt DC (2013). Longevity and mortality of owned dogs in England. Vet J.

[37] Diesel G, Brodbelt D, Laurence C (2010). Survey of veterinary practice policies and opinions on neutering dogs. Vet Rec.

[38] VetCompass. Demographic information on UK pets. 2015. http://www.rvc.ac.uk/vetcompass/infographics/uk. Accessed 20 May 2015.

[39] Looney AL, Bohling MW, Bushby PA, Howe LM, Griffin B, Levy JK (2008). The Association of Shelter Veterinarians veterinary medical care guidelines for spay-neuter programs. J Am Vet Med Assoc.

[40] The Cat Group. Policy statement 1: Timing of neutering. In: policy statements. 2006. http://www.thecatgroup.org.uk/policy_statements/neut.html. Accessed 20 May 2015.

[41] The Cat Group (2011). Cat neutering practices in the UK. J Feline Med Surg.

[42] Howe LM (1997). Short-term results and complications of prepubertal gonadectomy in cats and dogs. J Am Vet Med Assoc.

[43] Aronsohn MG, Faggella AM (1993). Surgical techniques for neutering 6- to 14-week-old kittens. J Am Vet Med Assoc.

[44] Olson PN, Kustritz MV, Johnston SD (2001). Early-age neutering of dogs and cats in the United States (a review). J Reprod Fertil Suppl.

[45] Spain CV, Scarlett JM, Houpt KA (2004). Long-term risks and benefits of early-age gonadectomy in cats. J Am Vet Med Assoc.

[46] Jones PH, Buchan I, Dawson S, Gaskell RM, Radford AD, Setzkorn C, et al. The social distribution of veterinary care. Society for Veterinary Epidemiology and Preventive Medicine (SVEPM); 28–30 March 2012; Glasgow (UK). 2012.

[47] Dohoo I, Martin W, Stryhn H, Dohoo I, Martin W, Stryhn H (2009). Introduction to observational studies. Veterinary epidemiologic research.

