# Supplementary material for: Demographics of dogs, cats, and rabbits attending veterinary practices in Great Britain as recorded in their electronic health records
Source: BMC Vet Res. 2017 Jul 11;13:218. doi: 10.1186/s12917-017-1138-9 (PMC5504643; doi:10.1186/s12917-017-1138-9)
Supplement: Supplementary file 1 — Demographics of the SAVSNET veterinary-visiting population of dogs, cats and rabbits by each region considered in the study. (DOCX 18 kb) [file 12917_2017_1138_MOESM1_ESM.docx]

|  |  | |  | |  |  | | |  | |  |  | | | |  | |  |  | | |  | |  |  |  | |  | |  | |  |  |
| --- | --- | --- | --- | --- | --- | --- | --- | --- | --- | --- | --- | --- | --- | --- | --- | --- | --- | --- | --- | --- | --- | --- | --- | --- | --- | --- | --- | --- | --- | --- | --- | --- | --- |
|  | Number of Species (%) | | | | | Age (median) | | | | Purebred (%) | | | | | Neutering (%) | | | | | Insurance (%) | | | | | | Microchipping (%) | | | | |  |  |  |
| Region | Dog | Cat | | Rabbit | | Dog | Cat | Rabbit | | Dog | | | Cat | Rabbit | Dog | | Cat | | Rabbit | Dog | Cat | | Rabbit | | | Dog | Cat | | Rabbit | |  |  |  |
| East Midlands | 8267 (67.2) | 3680 (28.6) | | 238 (1.9) | | 5.4 | 6.1 | 2.9 | | 85.2 | | | 9.3 | 100.0 | 58.8 | | 78.8 | | 44.1 | 17.5 | 8.0 | | 2.1 | | | 64.3 | 42.0 | | 3.8 | |  |  |  |
| East of England | 11332 (61.4) | 6205 (33.6) | | 436 (2.4) | | 5.3 | 6.1 | 2.9 | | 79.4 | | | 9.0 | 98.4 | 60.4 | | 76.2 | | 47.0 | 26.8 | 14.3 | | 3 | | | 59.0 | 45.6 | | 3.9 | |  |  |  |
| London | 2403 (49.5) | 2332 (48.1) | | 51 (1.1) | | 4.1 | 5.0 | 3.8 | | 83.7 | | | 12.5 | 100.0 | 62.5 | | 82.7 | | 51.0 | 39.6 | 30.5 | | 7.8 | | | 53.6 | 44.0 | | 0.0 | |  |  |  |
| North East | 16520 (72.7) | 4755 (20.9) | | 496 (2.2) | | 4.6 | 5.9 | 3.0 | | 82.9 | | | 10.6 | 99.1 | 49.4 | | 72.1 | | 45.4 | 24.3 | 15.8 | | 7.5 | | | 47.5 | 36.0 | | 5.4 | |  |  |  |
| North West | 28570 (64.2) | 13751 (30.9) | | 858 (1.9) | | 5.1 | 5.8 | 3.1 | | 83.6 | | | 9.8 | 99.2 | 56.6 | | 76.3 | | 46.9 | 28.4 | 19.3 | | 12.4 | | | 53.2 | 36.7 | | 7.1 | |  |  |  |
| South East | 32046 (59.4) | 19666 (36.5) | | 1173 (2.2) | | 5.4 | 6.6 | 2.7 | | 83.3 | | | 12.0 | 99.8 | 61.4 | | 78.9 | | 45.0 | 33.2 | 20.6 | | 8.3 | | | 58.2 | 46.6 | | 5.0 | |  |  |  |
| South West | 14648 (64.3) | 6871 (30.2) | | 488 (2.1) | | 5.5 | 6.5 | 3.0 | | 86.8 | | | 11.8 | 98.6 | 63.4 | | 81.1 | | 52.5 | 38.7 | 30.1 | | 36.5 | | | 58.6 | 45.0 | | 3.1 | |  |  |  |
| West Midlands | 12638 (64.2) | 6026 (30.6) | | 445 (2.3) | | 5.5 | 6.4 | 3.0 | | 86.2 | | | 10.1 | 91.8 | 58.8 | | 77.6 | | 44.5 | 26.5 | 17.4 | | 2.7 | | | 50.3 | 38.7 | | 2.0 | |  |  |  |
| Yorkshire and the Humber | 23490 (68.2) | 9477 (27.5) | | 616 (1.8) | | 4.9 | 6.4 | 3.3 | | 84.4 | | | 9.4 | 97.3 | 55.7 | | 76.7 | | 46.6 | 23.6 | 12.2 | | 2.4 | | | 55.5 | 39.5 | | 5.8 | |  |  |  |
|  |  |  |  |  |  |  |  |  |  |  |  |  |  |  |  |  |  |  |  |  |  |  |  |  |  |  |  |  |  |  |  |  |  |
| Scotland | 9153 (66.9) | 4004 (29.3) | | 231 (1.7) | | 5.4 | 6.7 | 3.8 | | 88.8 | | | 10.9 | 95.3 | 57.0 | | 76.0 | | 45.9 | 31.5 | 21.9 | | 4.3 | | | 43.6 | 32.1 | | 0.4 | |  |  |  |
| Wales | 15655 (71.6) | 5340 (24.4) | | 286 (1.3) | | 4.9 | 5.4 | 2.5 | | 84.2 | | | 9.5 | 97.9 | 48.8 | | 73.1 | | 40.9 | 16.4 | 9.2 | | 2.1 | | | 39.9 | 25.9 | | 2.4 | |  |  |  |
|  |  |  | |  | |  |  |  | |  | | |  |  |  | |  | |  |  |  | |  | | |  |  | |  | |  |  |  |

**Additional file 1**
