# Supplementary material for: Demographics of dogs, cats, and rabbits attending veterinary practices in Great Britain as recorded in their electronic health records
Source: BMC Vet Res. 2017 Jul 11;13:218. doi: 10.1186/s12917-017-1138-9 (PMC5504643; doi:10.1186/s12917-017-1138-9)
Supplement: Supplementary file 2 — Demographics of the SAVSNET veterinary-visiting population of dogs and cats summarised at practice level. (DOCX 13 kb) [file 12917_2017_1138_MOESM2_ESM.docx]

|  |  |  |
| --- | --- | --- |
| Demographics | Median of the all results obtained from each practice | Interval from all practices  (minimum value − maximum value) |
| Species |  |  |
| Dog | 66.2% | 34.1% − 85.4% |
| Cat | 29.9% | 13.1% − 54.6% |
| Age |  |  |
| Dog | 5.3 years | 2.6 − 7.8 years |
| Cat | 6.4 years | 2.3 − 10.1 years |
| Purebred |  |  |
| Dog | 83.9% | 57.1% − 94.0% |
| Cat | 9.6% | 2.9% − 42.1% |
| Neutering |  |  |
| Dog | 59.5% | 31.7% − 75.6% |
| Cat | 78.3% | 55.1% − 91.0% |
| Insurance |  |  |
| Dog | 27.3% | 1.3% − 99.9% |
| Cat | 14.3% | 0.5% − 99.0% |
| Microchipping |  |  |
| Dog | 56.0% | 24.7% − 87.4% |
| Cat | 40.5% | 8.1% − 70.4% |
|  |  |  |

**Additional file 2**
