# Supplementary material for: Demographics of dogs, cats, and rabbits attending veterinary practices in Great Britain as recorded in their electronic health records
Source: BMC Vet Res. 2017 Jul 11;13:218. doi: 10.1186/s12917-017-1138-9 (PMC5504643; doi:10.1186/s12917-017-1138-9)
Supplement: Supplementary file 3 — Number of animals stratified by species, British country and Index of Multiple Deprivation (IMD). Species assessed include dogs, cats and other species. IMD category 1 indicates the least deprived areas and category 5 the most deprived. The percentage that each species made up within each IMD category in each country is shown in brackets. (DOCX 13 kb) [file 12917_2017_1138_MOESM3_ESM.docx]

**Additional file 3**

| Country | IMD | Number of dogs (percentage) | Number of cats (percentage) | | Number of other species (percentage) |
| --- | --- | --- | --- | --- | --- |
| England | 5 | 18801 (66.4) | 8515 (30.1) | | 977 (3.5) |
|  | 4 | 23305 (63.8) | 11841 (32.4) | | 1384 (3.8) |
|  | 3 | 32823 (65.4) | 15530 (30.9) | | 1866 (3.7) |
|  | 2 | 40389 (66.3) | 18349 (30.1) | | 2201 (3.6) |
|  | 1 | 34945 (62.8) | 18521 (33.3) | | 2209 (3.9) |
| Wales | 5 | 1839 (75.0) | 526 (21.5) | | 87 (3.5) |
|  | 4 | 2761 (74.2) | 871 (23.4) | | 90 (2.4) |
|  | 3 | 4387 (70.5) | 1649 (26.5) | | 185 (3.0) |
|  | 2 | 4762 (72.3) | 1634 (24.8) | | 187 (2.9) |
|  | 1 | 869 (73.6) | 265 (22.5) | | 46 (3.9) |
| Scotland | 5 | 717 (69.5) | 281 (27.3) | | 33 (3.2) |
|  | 4 | 987 (66.5) | 455 (30.6) | | 43 (2.9) |
|  | 3 | 2049 (70.4) | 764 (26.2) | | 99 (3.4) |
|  | 2 | 2727 (68.9) | 1110 (28.0) | | 123 (3.1) |
|  | 1 | 2670 (63.6) | 1395 (33.2) | | 134 (3.2) |
|  |  | |  |  |  |
