# Supplementary material for: Demographics of dogs, cats, and rabbits attending veterinary practices in Great Britain as recorded in their electronic health records
Source: BMC Vet Res. 2017 Jul 11;13:218. doi: 10.1186/s12917-017-1138-9 (PMC5504643; doi:10.1186/s12917-017-1138-9)
Supplement: Supplementary file 4 — Results of the mixed effects logistic regression models, assessing the association between a range of an animal’s characteristics and the Index of Multiple Deprivation (IMD). Shown are odds ratios of fixed effects IMD in England, Wales and Scotland from the final mixed effects logistic regression models of; the probability of animals being a dog in the veterinary-visiting population; the probability of animals being a cat in the veterinary-visiting population; and of the probability of dogs and cats being purebred in the veterinary-visiting dog and cat population, respectively. Three asterisks (***), two asterisks (**) and one asterisk (*) indicate p < 0.001, p < 0.01 and p < 0.05, respectively. CI = confidence interval. (DOCX 15 kb) [file 12917_2017_1138_MOESM4_ESM.docx]

|  |  | Dog ownership: yes | | Cat ownership: yes | | | Breed ownership: purebred | | | |
| --- | --- | --- | --- | --- | --- | --- | --- | --- | --- | --- |
|  |  |  |  |  |  | | Dog | | Cat | |
| Country | IMD | Odds ratio (95% CI) | | Odds ratio (95% CI) | | | Odds ratio (95% CI) | | Odds ratio (95% CI) | |
| England | 5 | Reference | - | Reference | | - | Reference | - | Reference | - |
|  | 4 | 0.92 | (0.89 - 0.95)*** | 1.08 | | (1.05 - 1.12)*** | 1.04 | (0.98 - 1.09) | 1.05 | (0.94 - 1.17) |
|  | 3 | 0.95 | (0.92 - 0.98)** | 1.04 | | (1.01 - 1.08)* | 1.15 | (1.09 - 1.21)*** | 1.28 | (1.15 - 1.42)*** |
|  | 2 | 0.96 | (0.93 - 0.99)* | 1.04 | | (1.01 - 1.08)* | 1.23 | (1.16 - 1.30)*** | 1.46 | (1.31 - 1.62)*** |
|  | 1 | 0.89 | (0.85 - 0.92)*** | 1.12 | | (1.08 - 1.16)*** | 1.28 | (1.21 - 1.36)*** | 1.49 | (1.34 - 1.66)*** |
| Wales | 5 | Reference | - | Reference | | - | Reference | - | Reference | - |
|  | 4 | 0.88 | (0.78 - 0.99)* | 1.19 | | (1.04 - 1.35)** | 1.17 | (0.99 - 1.39) | 0.92 | (0.61 - 1.40) |
|  | 3 | 0.76 | (0.68 - 0.85)*** | 1.34 | | (1.19 - 1.51)*** | 1.06 | (0.90 - 1.24) | 1.35 | (0.93 - 1.95) |
|  | 2 | 0.80 | (0.71 - 0.90)*** | 1.26 | | (1.12 - 1.43)*** | 1.25 | (1.06 - 1.47)** | 1.32 | (0.90 - 1.95) |
|  | 1 | 0.88 | (0.74 - 1.04) | 1.08 | | (0.90 - 1.29) | 1.13 | (0.89 - 1.42) | 1.35 | (0.80 - 2.29) |
| Scotland | 5 | Reference | - | Reference | | - | Reference | - | Reference | - |
|  | 4 | 0.87 | (0.73 - 1.03) | 1.17 | | (0.98 - 1.41) | 0.92 | (0.68 – 1.25) | 1.40 | (0.81 - 2.43) |
|  | 3 | 0.90 | (0.76 - 1.06) | 1.09 | | (0.92 - 1.30) | 1.14 | (0.86 - 1.53) | 1.47 | (0.88 - 2.47) |
|  | 2 | 0.85 | (0.72 - 0.99)* | 1.18 | | (1.00 - 1.39)* | 1.19 | (0.90 - 1.57) | 1.51 | (0.92 - 2.50) |
|  | 1 | 0.80 | (0.69 - 0.94)** | 1.24 | | (1.06 - 1.45)** | 1.38 | (1.03 - 1.83)* | 1.53 | (0.94 - 2.50) |

**Additional file 4**
