# Supplementary material for: Demographics of dogs, cats, and rabbits attending veterinary practices in Great Britain as recorded in their electronic health records
Source: BMC Vet Res. 2017 Jul 11;13:218. doi: 10.1186/s12917-017-1138-9 (PMC5504643; doi:10.1186/s12917-017-1138-9)
Supplement: Supplementary file 5 — Results of the mixed effects logistic regression models, assessing the association between a range of an animal’s key performance indicators and the Index of Multiple Deprivation (IMD). Shown are odds ratios of fixed effects IMD in England, Wales and Scotland from the final mixed effects logistic regression models of; the probability of dogs and cats being neutered by sex; the probability of dogs and cats being insured; and of the probability of dogs and cats being microchipped. Asterisk (*) indicates p < 0.05. CI = confidence interval. (DOCX 19 kb) [file 12917_2017_1138_MOESM5_ESM.docx]

**Additional file 5**

|  |  | Neutering: yes | | | | | | | | Insurance: yes | | | | Microchipping: yes | | | |
| --- | --- | --- | --- | --- | --- | --- | --- | --- | --- | --- | --- | --- | --- | --- | --- | --- | --- |
|  |  | Male dogs | | Female dogs | | Male cats | | Female cats | | Dog | | Cat | | Dog | | Cat | |
| Country | IMD | Odds ratio (95% CI) | | Odds ratio (95% CI) | | Odds ratio (95% CI) | | Odds ratio (95% CI) | | Odds ratio (95% CI) | | Odds ratio (95% CI) | | Odds ratio (95% CI) | | Odds ratio (95% CI) | |
| England | 5 | 1.00 | - | 1.00 | - | 1.00 | - | 1.00 | - | 1.00 | - | 1.00 | - | 1.00 | - | 1.00 | - |
|  | 4 | 1.42 | (1.34 - 1.50)* | 1.40 | (1.32 - 1.48)* | 1.22 | (1.11 - 1.35)* | 1.21 | (1.11 - 1.32)* | 1.25 | (1.18 - 1.31)* | 1.18 | (1.08 - 1.29)* | 1.14 | (1.10 - 1.19)* | 1.14 | (1.07 - 1.21)* |
|  | 3 | 1.63 | (1.54 - 1.72)* | 1.64 | (1.55 - 1.74)* | 1.42 | (1.29 - 1.57)* | 1.42 | (1.30 - 1.55)* | 1.38 | (1.31 - 1.45)* | 1.26 | (1.16 - 1.38)* | 1.17 | (1.12 - 1.21)* | 1.21 | (1.14 - 1.28)* |
|  | 2 | 1.72 | (1.63 - 1.81)* | 1.84 | (1.74 - 1.95)* | 1.52 | (1.38 - 1.68)* | 1.53 | (1.40 - 1.68)* | 1.46 | (1.39 - 1.54)* | 1.40 | (1.28 - 1.52)* | 1.30 | (1.25 - 1.35)* | 1.22 | (1.15 - 1.30)* |
|  | 1 | 1.90 | (1.80 - 2.02)* | 2.19 | (2.06 - 2.33)* | 1.73 | (1.56 - 1.92)* | 1.67 | (1.52 - 1.84)* | 1.82 | (1.73 - 1.92)* | 1.50 | (1.37 - 1.65)* | 1.41 | (1.35 - 1.47)* | 1.38 | (1.29 - 1.46)* |
| Wales | 5 | 1.00 | - | 1.00 | - | 1.00 | - | 1.00 | - | 1.00 | - | 1.00 | - | 1.00 | - | 1.00 | - |
|  | 4 | 1.45 | (1.21 - 1.73)* | 1.33 | (1.11 - 1.59)* | 1.38 | (0.97 - 1.96) | 0.84 | (0.61 - 1.71) | 1.08 | (0.92 - 1.28) | 1.16 | (0.79 - 1.71) | 1.14 | (0.99 - 1.29) | 1.20 | (0.92 - 1.56) |
|  | 3 | 1.61 | (1.36 - 1.91)* | 1.69 | (1.42 - 2.00)* | 1.72 | (1.24 - 2.38)* | 1.12 | (0.82 - 1.52) | 1.36 | (1.16 - 1.58)* | 1.72 | (1.22 - 2.44)* | 1.32 | (1.17 - 1.49)* | 1.42 | (1.11 - 1.82)* |
|  | 2 | 1.75 | (1.47 - 2.08)* | 1.72 | (1.44 - 2.04)* | 1.86 | (1.32 - 2.62)* | 1.22 | (0.88 - 1.68) | 1.46 | (1.24 - 1.72)* | 1.48 | (1.01 - 2.16)* | 1.40 | (1.24 - 1.59)* | 1.26 | (0.97 - 1.63) |
|  | 1 | 2.09 | (1.64 - 2.66)* | 2.24 | (1.74 - 2.88)* | 1.81 | (1.08 - 3.03)* | 1.43 | (0.88 - 2.35) | 1.69 | (1.36 - 2.11)* | 1.90 | (1.16 - 3.12)* | 1.39 | (1.17 - 1.66)* | 1.46 | (1.02 - 2.07)* |
| Scotland | 5 | 1.00 | - | 1.00 | - | 1.00 | - | 1.00 | - | 1.00 | - | 1.00 | - | 1.00 | - | 1.00 | - |
|  | 4 | 1.26 | (0.96 - 1.65) | 1.73 | (1.29 - 2.34)* | 1.11 | (0.69 - 1.80) | 0.82 | (0.50 - 1.33) | 1.27 | (0.98 - 1.65) | 0.92 | (0.59 - 1.45) | 1.25 | (1.02 - 1.54)* | 0.95 | (0.68 - 1.33) |
|  | 3 | 1.32 | (1.03 - 1.70)* | 1.81 | (1.37 - 2.40)* | 1.19 | (0.76 - 1.88) | 1.05 | (0.65 - 1.69) | 1.42 | (1.12 - 1.81)* | 1.00 | (0.65 - 1.55) | 1.42 | (1.17 - 1.71)* | 1.16 | (0.84 - 1.59) |
|  | 2 | 1.46 | (1.14 - 1.86)* | 1.91 | (1.46 - 2.50)* | 1.25 | (0.81 - 1.94) | 1.00 | (0.64 - 1.59) | 1.43 | (1.35 - 1.81)* | 1.19 | (0.79 - 1.80) | 1.24 | (1.03 - 1.49)* | 1.34 | (0.99 - 1.82) |
|  | 1 | 1.62 | (1.27 - 2.06)* | 2.89 | (2.20 - 3.79)* | 1.85 | (1.20 - 2.86)* | 1.68 | (1.07 - 2.65)* | 2.11 | (1.68 - 2.65)* | 1.37 | (0.92 - 2.03) | 1.81 | (1.51 - 2.17)* | 1.40 | (1.04 - 1.89)* |
